# A pharmacological challenge paradigm to assess neural signatures of script-elicited acute dissociation in women with post-traumatic stress disorder

**DOI:** 10.1192/bjo.2023.34

**Published:** 2023-05-02

**Authors:** Yoki L. Mertens, Antje Manthey, Anika Sierk, Peter de Jong, Henrik Walter, Judith K. Daniels

**Affiliations:** Department of Clinical Psychology and Experimental Psychopathology, University of Groningen, Groningen, The Netherlands; Charité University Clinic Berlin (corporate member of Freie Universität Berlin, Humboldt-Universität zu Berlin and Berlin Institute of Health), Berlin, Germany

**Keywords:** PTSD, dissociation, biomarkers, reboxetine, imaging

## Abstract

**Background:**

There is limited experimentally controlled neuroimaging research available that could explain how dissociative states occur and which neurobiological changes are involved in acute post-traumatic dissociation.

**Aims:**

To test the causal hypothesis that acute dissociation is triggered bottom-up by a selective noradrenergic-mediated increase in amygdala activation during the processing of autobiographical trauma memories.

**Method:**

Women with post-traumatic stress disorder (*n* = 47) and a history of interpersonal childhood trauma underwent a within-participant, placebo-controlled pharmacological challenge paradigm (4.0 mg reboxetine versus placebo) employing script-driven imagery (traumatic versus neutral autobiographical memory recall). Script-elicited brain activation patterns (measured via functional magnetic resonance imagery) were analysed by means of whole-brain analyses and a pre-registered region of interest (i.e. amygdala).

**Results:**

Self-reported acute dissociation increased significantly during trauma (versus neutral) recall but did not differ between pharmacological conditions. The pharmacological manipulation was also unsuccessful in eliciting increased amygdala activation following script-driven imagery in the reboxetine (versus placebo) condition. In the reboxetine condition, trauma retrieval resulted in similar activation patterns as in the placebo condition (e.g. elevated brain activation in the middle occipital gyrus and supramarginal gyrus), albeit with different peaks.

**Conclusions:**

Current (null) findings cast doubt on the suggested role of the amygdala in subserving dissociative processing of trauma memories. Alternative pharmacological manipulation approaches (e.g. ketamine) and analysis techniques (e.g. event-related independent component analysis) might provide better insight into the spatiotemporal dynamics and network shifts involved in dissociative experiences and autobiographical trauma memory recall.

Dissociation refers to a range of experiences, from detachment (i.e. feeling separated from self or surroundings) and physical and emotional numbing to dissociative amnesia and identity confusion. In the context of post-traumatic stress disorder (PTSD), individuals experiencing classic PTSD symptoms (re-experiencing, avoidance and hyperarousal) as well as post-traumatic dissociation, such as derealisation and depersonalisation, are classified as belonging to the dissociative subtype. Research has reported that individuals diagnosed with the dissociative subtype depict decreased resting-state activation in limbic regions such as the amygdala,^1^ increased resting-state activation in frontal regions (predominantly the right ventromedial prefrontal cortex, orbitofrontal cortex and frontal pole) and elevated connectivity between the left basolateral amygdala and the insula,^2^ frontal and parietal regions^3^ and the periaqueductal grey.^4^ The ventrolateral part of the periaqueductal grey further depicted increased functional connectivity with the temporoparietal junction and rolandic operculum in individuals with dissociative PTSD.^5^ These brain regions are associated with multisensory processing, self–other distinction and depersonalisation experiences.^6,7^ Experimental functional magnetic resonance imagery (fMRI) studies (e.g. script-driven imagery, SDI) correlated task-elicited brain activation clusters with self-reported dissociation and found brain–behaviour correlates in frontal regions (increased^8,9^), temporal regions (increased and decreased^9^), insula (decreased^9^) and amygdala (decreased^10,11^). Taken together, these findings provide tentative support for the notion that altered frontolimbic circuitry may correspond to a pattern of overmodulated emotional reactivity between prefrontal regions and the amygdala in the dissociative subtype.^12^ However, nearly all available studies are correlational in nature. Bringing a central mechanism of dissociative processing under experimental control might open a window into analysing the underlying neural processes. Pharmacological interventions might offer one suitable approach to gaining experimental control over a fleeting subjective experience.

## Neuroendocrinological correlates of dissociative symptoms

The reciprocal disinhibition of the feedback loop between the locus ceruleus and the amygdala may serve as a possible neuroendocrinological substrate for PTSD. Previous research has shown that β-adrenergic stimulation of the amygdala is associated with increased reconsolidation of fear memory and impaired extinction learning,^13^ which may explain why intrusion symptomatology seems to be particularly severe and resistant to change in individuals diagnosed with the dissociative subtype. Neuropeptide Y (NPY), which regulates central noradrenaline release, is released centrally in the locus ceruleus, amygdala, hippocampus and periaqueductal grey. NPY receptors have been identified in the basolateral nucleus of the amygdala, and studies with knockout mice showed that the absence of these receptors is associated with deficient extinction learning.^14^ Increasing NPY levels in the amygdala have further shown an anxiolytic effect in animal studies.^15^ Several studies have provided evidence that post-traumatic dissociation is associated with dysregulation of NPY;^16–18^ specifically, adults with a history of childhood abuse who are carriers of a gene variant that leads to reduced production of NPY showed increased activation in the amygdala in response to emotive stimuli.^19^

It was therefore hypothesised that dissociative symptoms in individuals with PTSD during trauma exposure can be pharmacologically provoked by increasing noradrenaline concentrations. Initial studies reported that, whereas non-dissociative individuals with PTSD reported only intrusive re-experiencing under the pharmacological treatment, individuals with the dissociative subtype experienced additional dissociative reactions.^20,21^ Another pharmacological challenge study indicated that when noradrenaline concentration was significantly increased via the selective noradrenaline reuptake inhibitor reboxetine, the basolateral amygdala exhibited differential activation specifically in response to fearful, but not neutral facial expressions^22^ (replicated by^23^). Conversely, the pharmacological blockade of noradrenaline transmission via propranolol led to a reduced amygdala activation,^24^ supporting the hypothesis that increased noradrenaline concentration relates to increased amygdala activation and reactivity to threat cues in humans.

In sum, there are theoretical arguments^12^ as well as some evidence consistent with the view that alterations in the frontolimbic circuitry are involved in dissociative experiences and that increased amygdala excitability may underlie acute post-traumatic dissociation. It remains, however, to be tested whether an initial (noradrenergic-mediated) overactivation of the amygdala can indeed be seen as a causal factor in the occurrence of acute post-traumatic dissociation in response to idiosyncratic trauma recall in individuals with PTSD.

## Study aim

The current study aimed to test whether acute post-traumatic dissociation is related to increased reactivity of the amygdala during the recall of autobiographical trauma memories. We tested whether increased amygdala activation results in increased severity of dissociative responses when individuals with PTSD are exposed to a script of their traumatic memory. To bring individual amygdala activation levels under experimental control, we used reboxetine (a selective noradrenaline reuptake inhibitor) in the context of a within-participant placebo-controlled design. Although the naturally evocable dissociation occurs under the placebo condition (findings published in our previous paper^25^), a comparison of reboxetine versus placebo served to test the causal hypothesis that dissociation severity is a function of a selective noradrenergic-mediated increase in amygdala activation (i.e. the pre-registered region of interest).

## Method

### Participants

Participants were recruited by means of public advertisements and mental health treatment centres. Interested individuals were first screened via telephone for MRI compatibility and medication status. They were fully informed about the study procedure and potential risks before signing the informed consent. Written informed consent was obtained from all participants. They received a booklet containing the self-report instruments and were asked to complete these at home. At least one full day before the scanning session, interested individuals were invited to the laboratory to undergo clinical diagnostics. They were included in the study if they met all inclusion criteria: (a) 20–60 years of age, (b) proficient in German, (c) MRI compatible, (d) no neurological disorder, (e) no history of head injury, (f) no substance dependency in the past 6 months, (g) no intake of benzodiazepine or anticonvulsants, (h) PTSD as a primary disorder, (i) PTSD symptoms for at least 3 months, (j) experience of interpersonal childhood trauma before the age of 22 and (k) female. Only women were included to omit the need to control for gender in the analyses and to facilitate participant recruitment, as women are more likely to be diagnosed with PTSD. The age range (20–60 years) was chosen to reduce variance with regard to potential brain maturing effects above the age of 18 years (as well as allowing for a minimum of a 2-year age gap between the childhood traumatic events and the experiment) and atrophy effects already visible at the age of 60 years.^26^ To account for the high comorbidity encountered in PTSD, participants with the following comorbid disorders as a secondary diagnosis were eligible: depressive, anxiety, eating, substance use and borderline personality disorders. Individuals with other comorbidities (e.g. dissociative disorders) were excluded. Following data collection on 51 participants, 3 participants were excluded because of missing log files; 1 participant was removed because of missing data on the second testing session (reason: participant had an accident between the two days of scanning). The resulting sample included for analysis consisted of 47 female participants (mean age 40.07 years, s.d. = 10.10) diagnosed with PTSD.

### Procedure

The study inspected the within-participant effects of reboxetine versus placebo intake in females with PTSD. Participants were scanned twice and the order of medication intake was counterbalanced across participants. The procedure was double-blinded to prevent investigator effects, thus neither the participant nor the experimenter was aware of the condition for each scan. Participants underwent oral administration of 4.0 mg reboxetine (challenge condition: identical procedure to a previous publication^22^) or a placebo pill. Half of the participants received reboxetine on the first day and placebo on the second day, and vice versa (random allocation), with at least 72 h between the two days (this corresponds to five times the half-life (13 h) of reboxetine). Since the pharmacological agent reboxetine reaches maximum serum concentrations after 2 h,^27^ the scan was conducted 2 h after administration of the agent or placebo. In previous studies, a single administration of reboxetine led to only a few side-effects, even at a dosage twice as high as we had planned.^23^ The majority of reported side-effects were related to the visual and gastrointestinal systems; sleep was unaffected. Participants underwent a neuroimaging paradigm including an anatomical brain scan (in the first session only) and a functional paradigm called script-driven imagery. This paradigm comprised two condition blocks (a neutral condition block followed by a trauma condition block), each consisting three of consecutive runs (repetitions of the script presentation). Participants listened to autobiographical memory scripts (27 s) and were instructed to actively recall their traumatic incident (33.75 s) followed by a 2 min break (a detailed explanation of the procedure appears in our previous paper^25^). A successful pharmacological manipulation was defined *a priori* as increased amygdala activation in the main contrast of interest, i.e. ‘reboxetine (trauma versus neutral) > placebo (trauma versus neutral)'. As a secondary outcome measure, we tested whether reboxetine intake differentially affected subjective responding to trauma recall (i.e. acute script-elicited dissociation measured using the Response to Script-Driven Imagery Scale, RSDI^28^). Two previous publications^25,29^ are largely based on the same data-set.

The authors assert that all procedures contributing to this work comply with the ethical standards of the relevant national and institutional committees on human experimentation and with the Helsinki Declaration of 1975, as revised in 2008. The study was approved by the medical ethics board of the University of Magdeburg (57/14) and the ethical committee of the Berlin Psychological University and was pre-registered (aspredicted.org: #80954).

### Diagnostic and self-report measures

**Fig. 1:**
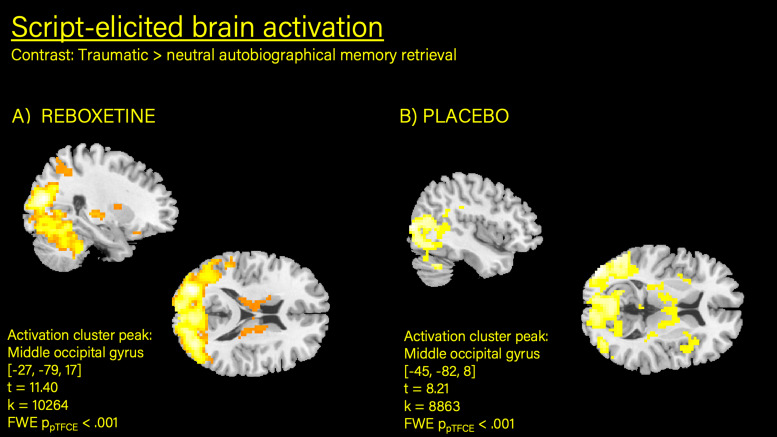
Increased brain activation detected in middle occipital gyrus during autobiographical memory retrieval (trauma > neutral).

Potential participants were screened and diagnosed using the German versions of the Structured Clinical Interview for DSM-IV for Axis I Disorders,^30^ the Structured Clinical Interview for Dissociative Disorders (SCID-D)^31^ and the Clinician-Administered PTSD Scale (CAPS-IV).^32^ Following their inclusion, participants completed various self-report questionnaires to assess traumatic experiences (Childhood Trauma Questionnaire,^33^ Essen Trauma Inventory^34^) as well as PTSD severity (Posttraumatic Stress Disorder Checklist^35^), trait dissociation (Fragebogens zu dissoziativen Symptomen [Dissociative Symptoms Questionnaire]^36^), depersonalisation (Cambridge Depersonalisation Scale-30^37^), peritraumatic dissociation (Peritraumatic Dissociative Experiences Questionnaire^38^), trait anxiety (State–Trait Anxiety Inventory – Trait Version^39^) and depression (Beck Depression Inventory-II^40^). In the two scanning sessions, participants indicated their level of state dissociation (Clinician-Administered Dissociative States Scale^41,42^) once before entering the MRI scanner and once immediately after exiting the scanner. At the end of each block (neutral and trauma) of the SDI, participants immediately rated their level of acute post-traumatic dissociation using the dissociation subscale of the RSDI.^28^ The subscale consisted of four items assessing acute derealisation and depersonalisation, each rated on a scale from 0 (‘not at all’) to 6 (‘a great deal’), with a higher rating indicating increased symptom severity experienced across the three runs. Additionally, a single dissociation item (‘During run X, did you experience detachment sensations (dissociation)?’) was assessed retrospectively to assess acute dissociation experienced in each run separately (for better visualisation of the study procedure, see Fig. 1 in our previous paper^25^).

### Data analysis

#### Script-evoked dissociative symptoms

To test whether the severity of acute dissociation experienced during script-elicited recall differed between the reboxetine and placebo conditions, a two-way repeated-measures analysis of variance (ANOVA) with condition (reboxetine versus placebo) and script (trauma versus neutral) was employed. Based on previous findings,^9,10^ we expected a main effect for script in the direction that exposure to the trauma (versus neutral) autobiographical script leads to increased dissociative experiencing. Furthermore, the main effect for condition was assessed to test whether reboxetine intake leads to increased dissociative experiencing as compared with placebo, and the interaction effect for condition × script was assessed to test whether dissociation elicited in the trauma script depended on the level of the condition (reboxetine versus placebo intake).

To test whether the repeated presentation of the autobiographical scripts affected symptom levels (e.g. potential habituation or sensitisation effects), the single dissociation item ratings were subjected to a three-way repeated-measures ANOVA, with condition (reboxetine versus placebo), script (trauma versus neutral) and time (first versus second versus third repetition) as within-participant factors. Finally, to test whether state dissociation (CADSS score) increased overall following the neuroimaging experiment and whether this differed between conditions, a two-way repeated-measures ANOVA with condition (reboxetine versus placebo) and assessment time (pre- versus post-experiment) was employed.

#### fMRI data acquisition

Participants were scanned at the Berlin Institute of Health, Charité – Universitätsmedizin Berlin, in a 3 T Siemens Magnetom TrioTim (*syngo*.MR; Siemens, Erlangen, Germany) equipped with a 12-channel hybrid birdcage radiofrequency coil for magnetic resonance signal transmission and reception. For the anatomical brain scan, high-resolution T1-weighted anatomic images were acquired using magnetisation-prepared rapid acquisition with a gradient echo sequence scanned in sagittal orientation (scanning parameters: field of view FoV = 256, 192 slices, 1 mm isotropic voxel size, repetition time TR = 1.9 s, echo time TE = 2.52 ms, flip angle 9°, time TI = 900 ms, 50% distancing factor). Functional images were scanned within a set of 40 contiguous, transversely oriented, 3-mm thick functional slices that were prescribed parallel to the anterior commissure–posterior commissure (AC–PC) plane. The T2*-weighted blood oxygen level-dependent (BOLD) functional brain volumes were obtained using gradient echo planar imaging (EPI) with an interleaved slice acquisition (64 × 64 matrix size, TR = 2.25 s, TE = 25 ms, flip angle 80°, FoV = 192 mm, distancing factor 20%).

### fMRI data analysis

#### fMRI preprocessing

Image processing and statistical analyses were performed using Statistical Parametric Mapping (SPM for Mac, version 12; Wellcome Department of Neurology, London, UK; https://www.fil.ion.ucl.ac.uk/spm/software/spm12/) within MATLAB 9.9.0 (R2020b; Math-Works for Mac; MathWorks, Inc., Natick, Massachusetts, USA; www.mathworks.com/products/matlab.html). Digital Imaging and Communications in Medicine (DICOM) images were converted to Neuroimaging Informatics Technology Initiative (NIfTI) format using the dcm2niix.mac software (version 2 for Mac, 3 November 2020; Chris Rorden, University of South Carolina, South Carolina, USA; https://github.com/rordenlab/dcm2niix/releases). Field map-derived voxel displacement maps (VDMs) were calculated per session to correct for motion distortions. The functional images corresponding to each script condition (trauma versus neutral imagery) were realigned and unwarped to the mean image per condition session. Consecutively, co-registration of the anatomical image to the unwarped mean image, segmentation of the co-registered anatomical image, normalisation (3 × 3 × 3 mm) of the realigned functional images to a Montreal Neurological Institute (MNI) anatomical template, and spatial smoothing to a Gaussian kernel of 6 mm full-width half-maximum (FWHM) took place. Field map data were missing for one participant, and here an alternative preprocessing pipeline without field map motion correction was applied. Visual inspection of the resulting preprocessed images did not indicate any meaningful divergences between pipelines.

#### First-level analysis

Voxel-wise general linear models (GLMs) were used to investigate the activation patterns during each condition. The following conditions were modelled as regressors: ‘retrieval’ (27 s) and ‘re-experiencing’ (33.75 s), present for the sessions ‘neutral’ and ‘trauma’. Conditions within the tasks were contrasted voxel-wise to each other in first-level analyses to identify areas that are less or more active in the trauma (versus neutral) condition at the participant level. Contrast maps for the contrast ‘trauma > neutral’ were calculated per participant for the whole recall period, as well as retrieval and re-experiencing, separately. The present study used the null periods before (30 s), in between runs (i.e. the 2 min rest period in between script presentation) and after (30 s) the SDI paradigm as the implicit baseline measures. During these periods, participants had their eyes open staring at a fixation cross and were instructed to relax and ‘let go’ of the memory material.

### Second-level analyses

#### Whole-brain analysis

Whole-brain analyses were employed to identify task-elicited activation clusters of the within-participant contrast ‘reboxetine (trauma > neutral) versus placebo (trauma > neutral)’. First-level contrast maps contrasting brain activation elicited during trauma (versus neutral) autobiographical memory recall per participant were entered into a paired-sample *t*-test model for second-level group analysis comparing reboxetine with the placebo condition. The main analysis focused on the whole recall period (60 s) across the script and imagery periods to allow for maximal statistical power. Following previous research suggesting activation differences in the retrieval (first 30 s) and re-experiencing (final 30 s) phases,^25,43^ we inspected these separately to investigate potential brain activation differences underlying autobiographical memory recall. Models were tested with probabilistic threshold-free cluster enhancement (pTFCE^44^) with family-wise error (FWE) correction for statistical thresholding and multiple comparisons correction (*P* < 0.05). If no voxels survived this conservative approach, results thresholded at *P* < 0.001 (uncorrected, cluster extent *k* ≥ 10) were to be reported. Potentially significant activation clusters were correlated with measures of script-evoked acute dissociation^28^ to identify neural correlates of stress-induced dissociative experiencing.

#### Region of interest (ROI) approach

Testing the causal hypothesis that dissociation is steered by bottom-up noradrenergic pathways and thus is increased during reboxetine intake, amygdala activation was expected to be higher in the reboxetine versus the placebo condition. We defined and extracted the bilateral amygdala complex as described in the Julich Brain Atlas^45^ employing the JuBrain SPM Anatomy Toolbox for Mac, version 3.0 (Institute of Neuroscience and Medicine (INM-1, INM-7), Forschungszentrum Jülich, Jülich, Germany^46^). Small-volume correction (SVC) was applied to account for multiple testing and significant activation clusters correlated with dissociation measures.

## Results

Demographic variables for the participants are shown in Table 1. Descriptive statistics are shown in Table 2.

**Table 1:** Demographic variables for the study population (*n* = 47)

	% (*n*)	Mean (s.d.)
Age		40.70
School education (highest degree)		
High school diploma	68.1 (32)	
Junior high school diploma	31.9 (15)	
Professional training (highest degree)		
University degree	42.6 (20)	
Vocational training/apprenticeship	34.1 (16)	
Trainee	12.8 (6)	
None	10.6 (5)	
Relationship status^a^		
Single	51.1 (24)	
In a partnership	27.7 (13)	
Married	21.3 (10)	
Divorced	21.3 (10)	
Widowed	2.1 (1)	
Lifetime traumatic experiences (ETI-TL)^a^		
Childhood sexual abuse^b^	83.0 (39)	
Sexual abuse in adulthood^b^	55.3 (26)	
Violent attack^b^	76.6 (36)	
Neglect	66.0 (31)	
Severe illness	59.6 (28)	
Death of close person	51.1 (24)	
Accidental trauma	46.8 (22)	
Torture	19.1 (9)	
Natural disaster	19.1 (9)	
Imprisonment	12.8 (6)	
War combat	4.3 (2)	
Age at onset of worst traumatic experience, years		17.93 (12.81)
Interpersonal Childhood Trauma (CTQ)^c^		
Childhood sexual abuse (≥8)	72.3 (34)	13.87 (7.33)
Childhood physical abuse (≥8)	61.7 (29)	11.20 (5.81)
Childhood emotional abuse (≥10)	83.0 (39)	18.80 (5.65)
Childhood emotional neglect (≥15)	78.7 (37)	18.31 (4.84)
Childhood physical neglect (≥8)	76.6 (36)	11.53 (4.85)
Above cut-off on any abuse subscale	95.7 (45)	
Above cut-off on any neglect subscale	95.7 (45)	
Traumatic memory (used for SDI script)^d^		
Childhood trauma		
Childhood sexual abuse	55.3 (26)	
Childhood physical abuse	8.5 (4)	
Childhood combined sexual and physical abuse	10.6 (5)	
Other	4.3 (2)	
Adult trauma		
Adult sexual abuse	6.4 (3)	
Adult physical abuse	8.5 (4)	
Other	6.4 (3)	
Comorbid diagnoses (SCID-I and II)		
Anxiety disorder	17.0 (8)	
Affective disorders	21.3 (10)	
Borderline personality disorder	14.9 (7)	
Eating disorders	10.6 (5)	
Substance misuse	14.9 (7)	

ETI-TL, Essen Trauma Inventory – Trauma Checklist; CTQ, Childhood Trauma Questionnaire; SDI, script-driven imagery; SCID, Structured Clinical Interview for DSM-IV Axis I and Axis II Disorders; Mean, mean of the subscale sumscore; s.d., standard deviation.

a. Multiple answers were possible.

b. Perpetrated by a familiar person (e.g. family member), stranger or both.

c. Cut-offs in parentheses as established by Walker and colleagues (1999).^47^

d. Defined as the traumatic memory eliciting the strongest intrusion symptoms at assessment.

**Table 2:** Descriptive statistics for the study population (*n* = 47)

	*n*	Mean	s.d.	Range	Minimum	Maximum	Skewness statistic (s.d.)	Kurtosis statistic (s.d.)
Reboxetine acute dissociation (RSDI)	47	2.50	1.442	6	0	6	0.315 (0.347)	−0.406 (0.681)
Reboxetine acute re-experiencing (RSDI)	47	4.91	1.041	6	0	6	−1.255 (0.347)	2.306 (0.681)
Reboxetine acute avoidance (RSDI)	47	2.40	1.595	5	0	5	−0.024 (0.347)	−0.989 (0.681)
Reboxetine state dissociation (CADSS)	47	59.85	52.458	173	0	173	−2.444 (0.347)	9.811 (0.681)
Trait dissociation (FDS)	47	27.63	16.136	62	3	65	−0.049 (0.347)	−1.103 (0.681)
Depersonalisation (CDS)	44	69.98	49.664	203	0	203	0.939 (0.347)	−0.195 (0.681)
Somatoform dissociation (SDQ)	44	33.95	11.694	57	21	78	0.607 (0.347)	−1.018 (0.681)
Peritraumatic dissociation (PDEQ)	44	22.41	9.881	39	1	40	0.545 (0.347)	−0.403 (0.681)
Childhood trauma (CTQ)	45	73.71	22.227	88	28	116	0.835 (0.357)	0.197 (0.681)
Self-reported PTSD (PCL)	47	37.87	6.775	27	23	50	1.623 (0.357)	3.437 (0.681)
Interview-assessed PTSD severity (CAPS-IV)	47	67.55	14.365	55	40	95	−0.102 (0.357)	−0.982 (0.681)
Depression (BDI-II)	45	22.11	13.308	52	1	53	−0.090 (0.354)	−0.790 (0.681)
Trait anxiety (STAI-T)	44	55.41	10.400	38	37	75	−0.143 (0.347)	−0.545 (0.681)

RSDI, Responses to Script-Driven Imagery Scale; CADSS, Clinician- Administered Dissociative States Scale; FDS, German Version of the Dissociative Experiences Scale; CDS, Cambridge Depersonalisation Scale; SDQ, Somatoform Dissociation Questionnaire; PDEQ, Peritraumatic Dissociative Experiences Questionnaire; CTQ, Childhood Trauma Questionnaire; PTSD, post-traumatic stress disorder; PCL, PTSD Checklist for DSM-IV; CAPS-IV, Clinician-Administered PTSD Scale – Version IV; BDI-II, Beck Depression Inventory-II; STAI-T, State–Trait Anxiety Inventory – Trait Version.

### Severity of acute dissociation

Acute dissociation (RSDI dissociation subscale^28^) differed significantly depending on whether participants listened to and recalled the neutral or trauma script, with stronger acute dissociation elicited in the trauma script (two-way repeated measures ANOVA: significant main effect for script, *F*(1, 45) = 73.92, *P* < 0.001). However, results did not yield a main effect for condition (*F*(1, 45) = 0.31, *P* = 0.584), nor a significant interaction mean effect for condition × script (*F*(1, 45) = 0.81, *P* = 0.372). Thus, as predicted, acute dissociation was significantly elevated in the trauma (versus neutral) script presentation, but contrary to expectations this effect was not especially pronounced in the reboxetine condition.

#### Repetition effect

A three-way repeated measures ANOVA did not result in a significant three-way interaction of condition × script × repetition (*P =* 0.683), but indicated two significant two-way interactions for condition × script (*F*(1, 42) = 4.81, *P* = 0.034) and script × repetition (*F*(1.64, 68.77) = 6.22, *P* = 0.003; (Greenhouse–Geisser corrected results reported owing to sphericity violation). In the first interaction (condition × script), acute dissociation following the trauma script slightly increased from placebo to reboxetine, whereas the reverse pattern took place for the neutral script. However, pairwise comparisons (Bonferroni correction applied) did not find a significant difference in script presentation depending on the pharmacological condition. In the second interaction (script × repetition), dissociation levels significantly increased in response to repeatedly hearing the trauma script as opposed to the neutral condition, in which dissociation remained low across repetitions, independent of the pharmacological condition. Therefore, there was a significant main effect for script (*P* < 0.001) and run (*P* = 0.002), but not for condition (*P* = 0.840).

#### State dissociation

Two-way ANOVA of state dissociation levels assessed before and after the neuroimaging experiment showed that participants reported a significant increase in state dissociation following the experiment (*F*(1, 45) = 34.24, *P* < 0.001). Although a trend indicating a steeper increase in the reboxetine condition could be observed, neither the main effect of condition nor the interaction effect condition × time was statistically significant. Hence, changes in state dissociation did not appear to depend on whether the reboxetine or placebo pill was administered.

#### Neuroimaging findings

Whole-brain analysis of the main contrast of interest to test for brain activation differences following reboxetine (versus placebo) intake in a within-participant design ‘reboxetine (trauma > neutral) > placebo (trauma > neutral)’ at group level did not yield any significant clusters for the recall period at the pTFCE FWE-corrected threshold (nor at an uncorrected level of *P* < 0.001). Similarly, separate inspection of the retrieval (i.e. script presentation) and re-experiencing (i.e. imagery) periods did not result in significant differences between the two conditions.

Owing to the within-participant design used in this study, it is not useful to compute a main effect for script (trauma versus neutral) across both conditions. But when inspected separately, the contrast ‘trauma versus neutral recall’ within the reboxetine condition (one sample *t*-test) showed a large activation cluster in the bilateral occipital gyri (−27, −79, 17; *t* = 7.66, *z* = 6.12, FWE ppTFCE < 0.001, *k* = 3887; Table 3). Similarly, a large activation hub could be identified for the placebo condition (previously published^25^) centred on the cerebellum (−6, −70, −25; *t* = 7.25; *z* = 5.97; FWE ppTFCE < 0.001; *k* = 4236), including the inferior occipital lobe and vermis (Fig. 1). Inspection of the contrast ‘trauma versus neutral’ in the first 30 s of the recall period (i.e. retrieval) in the reboxetine condition showed robust activation in the left middle occipital (−27, −79, 17; *t* = 11.40, *z* = 7.81, FWE ppTFCE < 0.001) and right supramarginal gyrus (57, −37, 29), right middle cingulate cortex,^3,10,44^ the left temporal pole of the middle temporal gyrus (−54, 11, −4), inferior frontal gyrus, insula, precentral gyrus and putamen. Similar brain activation clusters (with different activation peaks) were detected in the placebo condition.^25^ Direct statistical comparison of the two conditions via paired-sample *t*-test did not show significant activation differences. Regarding the second part of the recall period (i.e. re-experiencing), no significant activation clusters were identified for the contrast ‘trauma > neutral’ with FWE pTFCE statistical thresholding in the reboxetine condition (or in the placebo condition^25^). At an uncorrected level of *P* < 0.001, the imagery period in both conditions elicited scattered activation in similar brain regions of the occipital gyrus, cerebellum and cingulate.

**Table 3:** Script-elicited brain activation following the administration of reboxetine (4.0 mg) in *n* = 47 women with post-traumatic stress disorder^a^

MNI coordinates			*T*	*P* (FWE)	*k*	Automated anatomical label	Hemisphere
*x*	*y*	*z*					
Reboxetine: traumatic > neutral recall (retrieval + re-experiencing; 60 s)							
−27	−79	17	7.66	<0.000	3887	Middle occipital gyrus	Left
−6	−76	8	7.63	<0.000			
6	−91	5	7.55	<0.000			
−60	−43	32	5.79	0.013	12	Supramarginal gyrus	Left
−60	−52	14	5.65	0.020	52	Middle temporal gyrus	Left
−54	−64	20	5.54	0.027			
Reboxetine: traumatic > neutral retrieval (script presentation; first 30 s)							
−27	−79	17	11.40	<0.000	10264	Middle occipital gyrus	Left
30	−76	20	10.77	<0.000			
36	−82	11	10.49	<0.000			
57	−37	29	6.34	0.002	121	Supramarginal gyrus	Right
63	−40	35	6.05	0.006			
51	−31	23	5.91	0.010			
3	−10	44	6.12	0.005	77	Middle cingulate cortex	Right
−3	2	47	5.70	0.018			
−3	−16	50	5.60	0.024			
−54	11	−4	5.86	0.011	39	Superior temporal pole	Left
−57	2	8	5.65	0.021			
−54	8	−13	5.46	0.036			
−9	−7	71	5.84	0.012	29	Supplementary motor area	Left
0	−16	68	5.66	0.020			
51	32	−1	5.81	0.013	39	Inferior frontal gyrus, triangular part	Right
42	29	−1	5.79	0.014			
57	26	5	5.57	0.027			
−30	20	−19	5.72	0.017	48	Insula	Left
−30	29	−1	5.70	0.018			
−33	23	−10	5.67	0.020			
−45	−7	47	5.64	0.022	19	Precentral gyrus	Left
21	−22	68	5.61	0.023	13	Precentral gyrus	Right
27	−13	5	5.46	0.036	14	Putamen	
33	−16	−4	5.37	0.046			
Reboxetine: traumatic > neutral re-experiencing (imagery; final 30 s)^b^							
−	−	−	−	−	−	−	−

MNI, Montreal Neurological Institute; FWE, family-wise error; pTFCE, probabilistic threshold-free cluster enhancement.

a. Coordinates and anatomical labels for overactivations during script presentation and imagery for an FWE-corrected threshold of pTFCE < 0.05, with a cluster extent threshold of *k* ≥ 10.

b. No statistically meaningful findings.

### Amygdala activation (ROI)

To further inspect the causal hypothesis that dissociation severity is a function of a selective noradrenergic-mediated increase in amygdala activation (i.e. the pre-registered ROI), we employed small-volume correction for the bilateral amygdala across the whole recall period, as well as each processing phase (i.e. retrieval only and re-processing only) separately. Neither the left nor the right amygdala exhibited significantly increased or decreased activation when testing the main contrast of interest, ‘reboxetine (trauma > neutral) > placebo (trauma > neutral)’. In the reboxetine-only condition, amygdala activation did not significantly differ across the whole recall period (60 s). When inspecting only the initial 30 s (i.e. retrieval) in the reboxetine condition, increased activation in the right amygdala (24, −1, −13; *t* = 4.16, *k* = 3, SVC *P* = 0.005) was found (but no alterations in the left amygdala). For comparison, in the placebo condition only, elevated activity was found in the left amygdala (−33, −7, 19, *t* = 4.98, *k* = 21, SVC *P* < 0.001) and right amygdala (24, −4, −16, *t* = 3.87, *k* = 15, SVC *P* = 0.010^25^). For re-experiencing, similar to previously published findings for the placebo condition,^25^ no activation differences in the bilateral amygdala were derived during the final 30 s of trauma (versus neutral) recall.

### Dissociation brain–behaviour correlates

Additionally, we repeated the model with acute dissociation difference scores (trauma > neutral) as a covariate of interest per participant and condition, for the recall (and retrieval and re-experiencing) phase. Acute dissociation did not correlate significantly with brain activation between conditions following the main contrast of interest for recall, nor for retrieval and re-experiencing inspected separately.

## Discussion

The present study was designed to test whether a causal link exists between noradrenergic-mediated reactivity of the amygdala and the severity of dissociation, employing a pharmacological manipulation design. Forty-seven females with PTSD received 4.0 mg of reboxetine (a selective noradrenaline reuptake inhibitor) or a placebo (within-participant design; counterbalanced) before undergoing autobiographical memory recall (i.e. script-driven imagery) inside an MRI scanner to measure functional brain activation patterns. It was hypothesised that reboxetine intake – via increased noradrenaline concentration – would lead to differentially increased initial activation of the amygdala.

The major findings can be summarised as follows. The severity of acute post-traumatic dissociation was significantly stronger in response to the trauma versus the neutral script across conditions but did not differ between reboxetine versus placebo (neither as a main effect nor as an interaction with script type). Dissociation increased with the repetition of the script, indicating a sensitisation effect. Regarding neural activation, increased brain activation in the occipital/cerebellum region was observed during the processing of the trauma versus the neutral script. This was true for both conditions (reboxetine and placebo) separately (with a slight difference in foci). However, no significant differences in brain activation between the reboxetine and the placebo condition could be detected (either at the whole-brain level or in the ROI analyses of the bilateral amygdala). As our previous analyses^25^ indicated that the activation differences for the contrast ‘trauma versus neutral script’ stem mostly from the initial recall, i.e. the first 30 s of the recall period when the audio script was being played back to the patient, we opted to explore whether this was any different in the reboxetine condition. When inspecting the reboxetine condition separately, a difference in activation patterns between the first and the second part of the script-driven imagery paradigm was identified: during retrieval of trauma memories (i.e. script presentation), elevated brain activation in widespread brain regions including the middle occipital gyrus, supramarginal gyrus, middle cingulate gyrus, superior temporal pole and insula as well as the right amygdala was elicited as compared with listening to scripts of neutral autobiographical memories; conversely, during pure imagery (i.e. re-experiencing) no significant differences between the trauma versus neutral script conditions were identified. This is in line with what we reported earlier for the placebo condition.^25^

In sum, the current study failed to find a significant effect of the pharmacological condition (reboxetine versus placebo) on neural activation and subjective severity of dissociation elicited by the script-driven imagery paradigm. Therefore, the pharmacological manipulation to elicit increased amygdala activation was unsuccessful, which precluded the possibility of testing our causal hypothesis regarding the pivotal role of amygdala activation in the emergence of dissociative processing.

### Amygdala – an inconsistent functional neural correlate of dissociation

Across recent neuroimaging research and meta-analytic reviews,^48–50^ the assumed role of the amygdala as a neural marker of dissociation has come into question. Within the amygdala complex, the prominent basolateral amygdala is formed by the locus ceruleus and is noradrenergic innervated. In turn, the neurons of the locus ceruleus are triggered by strong emotional stimuli and subsequently lead to a noradrenergic-mediated increase in amygdala activation.^22^ The central nucleus of the amygdala sends projections to the ventromedial prefrontal cortex, the reticular formation and the periaqueductal grey. The ventrolateral nucleus of the periaqueductal grey is predominantly responsible for the interruption of motor impulses, which can result in immobility coupled with high muscle tone/tension.^51^ Additionally, the periaqueductal grey is part of the opioidergic system and modulates pain sensitivity as well as heart rate via descending pathways with the rostral ventromedial medulla and locus ceruleus. Considering the established role of the amygdala in emotional reactivity^52^ and the processing of negative stimuli,^53^ as well as the periaqueductal grey's involvement in modulating physical reactions (e.g. freezing or analgesia^5,51^), structural and functional aberrations in these subcortical areas have been hypothesised to play a key role in the development of dissociative experiences. Although singular findings point towards alternating amygdala activation, for instance in individuals with dissociative PTSD reacting to subliminal versus supraliminal threat stimuli,^52^ the majority of studies either present contradictory results or fail to find any meaningful alterations.^25,48^ A recent multi-centre investigation by Lebois and colleagues^50^ measured and analysed resting-state and functional fMRI data in over 1000 participants. Persistent derealisation measured 2 weeks post-trauma predicted 3-month PTSD symptom severity and was associated with decreased connectivity between the ventromedial prefrontal cortex and the cerebellum and orbitofrontal cortex. Notably, the same study was – similar to current null findings – unable to demonstrate a link between dissociation and amygdala (or insula) activation following an fMRI emotional reactivity task.^50^ Another resting-state analysis in a large sample with PTSD (*n* = 275) found decreased default mode network connectivity between the posterior cingulate cortex and right isthmus in relation to the severity of depersonalisation and derealisation, potentially indicating altered processing in sensory integration, consciousness and spatial awareness in the dissociative subtype of PTSD.^54^ Again, no meaningful activation alterations were reported regarding the amygdala. Taken together, recent findings based on resting-state data collected in large samples^50,54^ as well as current functional neuroimaging in a robust sample for fMRI data analyses (*n* > 30)^55^ cast doubt on the conceptualisation of acute post-traumatic dissociation as an exaggerated emotion overmodulation response elicited by initially elevated amygdala excitability.

### Reconsidering the blocked fMRI script-driven imagery paradigm

Alternatively, it is conceivable that study design and pre-registered analysis techniques were unsuited to scrutinising changes in amygdala activation, for example if one hypothesises a shift from an initial overactivation to hypoactivation in response to idiosyncratic trauma recall. First, SDI is a blocked fMRI design, which implicitly assumes relatively stable activation patterns as well symptomatology across periods of 60 s. However, recent reviews^43,56^ have suggested that two distinct processing stages underlying (traumatic) autobiographical memory can be identified, namely memory retrieval (increased cognitive recruitment warranted to activate memory association networks) and reprocessing (increased emotionality due to overwhelming intrusive imagery). Analysing the two parts of the SDI paradigm (i.e. listening to the script and imagery) separately, we identified starkly different brain activation patterns in the sense that the specific activation elicited by the trauma versus the neutral script was only observable during the initial phase. However, whether blocks of 30 s are the ideal time interval to interrogate such brain responses remains questionable. Some previous studies seem to indicate that regulation processes at the level of the amygdala might be much faster. Previous research by Lamke and colleagues^53^ assessing task–rest interactions in healthy adults undergoing an affective valence fMRI task found an early signal intensity peak at the beginning of unregulated emotional stimulation. If the participants were instructed to downregulate their subjective emotional reaction, bilateral amygdala activation was reduced during the negative valence stimulus presentation, but subsequently increased during rest – a process called the ‘amygdala rebound effect’ (previously detected by Walter et al^57^). Lamke et al assume this to represent ‘the amygdala's role as an alarm system which is suppressed during regulation’.^53^ Something similar might be at play when it comes to the initialisation of dissociative processing and might have precluded us from finding any differences between conditions and from gaining experimental control of amygdala activation following reboxetine intake. Employing, for example, a sliding window approach or event-related independent component analysis to assess spatiotemporal dynamics across the autobiographical recall period might be better suited to capturing rapid network shifts over the time course.^58^

### Pharmacological agents to investigate or elicit acute dissociation

Reboxetine is a selective noradrenaline reuptake inhibitor originally praised for its potency and limited side-effects for the treatment of depression or panic disorder; however, it has subsequently been suggested that a potential publication bias may have distorted perceptions of the antidepressant regarding the efficacy and remission rates.^59^ Unlike the results of Onur and colleagues,^22^ our neuroimaging data did not support reboxetine as a useful pharmacological agent for experimentally inducing elevated amygdala reactivity to investigate neural underpinnings of acute post-traumatic dissociation. From a neurobiological standpoint, the intake of 4.0 mg of reboxetine may have affected the basal metabolism of the amygdala, but we were unable to observe these changes with fMRI. Possibly, a higher dose (for instance, 8.0 mg as tested by Brühl et al^23^) is needed to reliably detect its effect on amygdala activation. In the following, we will briefly discuss ketamine, a non-competitive *N*-methyl-d-aspartate glutamate receptor (NMDAR) antagonist, as an alternative pharmacological agent to study dissociation. Across studies and populations (e.g. healthy, depressed, post-traumatic samples), ketamine (common dose: 0.5 mg/kg over 40 min) reliably induces dissociative experiences,^60,61^ which appear psychometrically similar to, but less intense than,^61^ naturally occurring dissociation in trauma-exposed samples. A study by Danboeck and colleagues^62^ measured alterations in resting-state functional connectivity following ketamine-induced dissociation in PTSD. Opposed to pre-registered hypotheses based on the dissociation model of emotion overmodulation by Lanius and colleagues^12^, experimentally induced dissociation resulted in decreased frontolimbic connectivity in individuals with PTSD receiving ketamine (*n* = 12) compared to the control drug (*n* = 14). Future research needs to determine how ketamine-induced modulation of neurotransmitter systems (possibly mediated by glutamatergic dysfunction) differs from naturally occurring trauma-related dissociation, which has been linked to the opioid system.^62^ Here, a comparative fMRI script-driven imagery design (ketamine versus placebo) analysed with event-related independent component analysis may provide new insights into experimentally elicited shifts in neural networks associated with acute post-traumatic dissociation as a reactive shutdown response to trauma reminders.

### Limitations

Several limitations need to be acknowledged. (a) No group comparison between classic PTSD and its dissociative subtype was conducted. Although group comparison was considered, we decided on a continuous, correlational approach that allowed us to test whether individual severity scores on dissociation measures correlated with elevated or decreased activation patterns (e.g. in the amygdala) for the following reasons. The clinical make-up of the current sample is much less clear-cut than theoretically conceptualised and dichotomising participants into groups based on scoring of interview and self-report measures turned out to be very difficult (see group allocation procedure in Sierk and colleagues,^29^ partly based on the same data-set). Moreover, membership in a potential dissociative subtype group did not predict acute dissociation experienced during trauma exposure in the scanner. The dichotomisation of the current sample into distinct profiles, next to the concurrent reduction of statistical power, thus did not seem sensible, given the ambiguous response patterns across diagnostic and self-report scales (see Table 2 in our previous paper^25^). (b) The current study did not include a trauma-exposed control group; this would have allowed it to specify whether the identified brain activation clusters are idiosyncratic to PTSD symptomatology. As suggested by Patel and colleagues^63^ and a recently published systematic review,^49^ structural and functional aberrations in the amygdala may be less related to the PTSD diagnosis or pathological dissociation and more related to trauma exposure itself. Here, longitudinal neuroimaging data, assessed pre- and post-trauma, is needed to gain clarity on the question of aetiological causal pathways. (c) The study did not assess or control for the menstrual cycle and whether participants were pre- or post-menopause. Research suggests that the menstrual cycle can affect women's severity of re-experiencing traumatic memories. For instance, women are almost five times as likely to experience flashbacks when in the luteal phase of the menstrual cycle, associated with increased glucocorticoid release,^64^ possibly inducing confounding effects in (neural) response patterns. (d) The selected sample was homogeneous in characteristics owing to a shared history of interpersonal childhood trauma and female gender, but varied in age, time since trauma onset and the frequency of other traumatic life events (Table 1). This heterogeneity may partially account for the inter-participant variability of neurobehavioural responses observed at the participant level, which may have led to the neutralisation of measured (e.g. increased and decreased) brain activation in relevant clusters, resulting in null effects across the averaged group level. Despite these shortcomings, concerted efforts were taken to reduce possible confounding due to repetition or anticipation effects, and to have a robust sample size less susceptible to type I and Type II errors^65^ by employing a within-participant, placebo-controlled, double-blind design with a randomised counterbalanced order of the reboxetine administration in a severely traumatised sample of *n* = 47 individuals with PTSD.

## Data Availability

The data that support the findings of this study are available on request from the corresponding author (J.K.D.). The data are not publicly available because they containing information that could compromise the privacy of research participants.
